# Improving public cancer care by implementing precision medicine in Norway: IMPRESS-Norway

**DOI:** 10.1186/s12967-022-03432-5

**Published:** 2022-05-14

**Authors:** Åslaug Helland, Hege G. Russnes, Gro Live Fagereng, Khalid Al-Shibli, Yvonne Andersson, Thomas Berg, Line Bjørge, Egil Blix, Bodil Bjerkehagen, Sigmund Brabrand, Marte Grønlie Cameron, Astrid Dalhaug, Dalia Dietzel, Tom Dønnem, Espen Enerly, Åsmund Flobak, Sverre Fluge, Bjørnar Gilje, Bjørn Tore Gjertsen, Bjørn Henning Grønberg, Kari Grønås, Tormod Guren, Hanne Hamre, Åse Haug, Daniel Heinrich, Geir Olav Hjortland, Eivind Hovig, Randi Hovland, Ann-Charlotte Iversen, Emiel Janssen, Jon Amund Kyte, Hedda von der Lippe Gythfeldt, Ragnhild Lothe, Jo-Åsmund Lund, Leonardo Meza-Zepeda, Monica Cheng Munthe-Kaas, Olav Toai Duc Nguyen, Pitt Niehusmann, Hilde Katarina NilsenPuco, Anne Hansen Ree, Tonje Bøyum Riste, Karin Semb, Eli Sihn Samdal Steinskog, Andreas Stensvold, Pål Suhrke, Øyvind Tennøe, Geir E. Tjønnfjord, Liv Jorunn Vassbotn, Eline Aas, Kristine Aasebø, Kjetil Tasken, Sigbjørn Smeland

**Affiliations:** 1 Institute for Cancer Research/Department of Oncology /Division of Cancer Medicine, Oslo University Hospital, Oslo, Norway; 2 Institute of Clinical Medicine, University of Oslo, Oslo, Norway; 3 Department of Pathology, Oslo University Hospital, Oslo, Norway; 4 Dept. of Pathology, Nordland Hospital, Bodø, Norway; 5 Hospital Pharmacies Enterprise, Oslo, Norway; 6 Department of Pathology, University Hospital in North of Norway, Tromsø, Norway; 7 Department of Clinical Medicine, UiT The Arctic University of Norway, Tromsø, Norway; 8 Haukeland University Hospital, Bergen, Norway; 9 Centre for Cancer Biomarkers CCBIO, Department of Clinical Science, University of Bergen, Bergen, Norway; 10 Department of Oncology, University Hospital in North of Norway, Tromsø, Norway; 11 Hospital of Southern Norway, Kristiansand, Norway; 12 Department of Oncology and Palliative Medicine, Nordland Hospital Trust, Bodø, Norway; 13 Telemark Hospital Trust, Skien, Norway; 14 Department of Research, The Cancer Registry of Norway, Oslo, Norway; 15 Department of Oncology, The Cancer Clinic, St Olavs Hospital, Trondheim University Hospital, Trondheim, Norway; 16 Department of Clinical and Molecular Medicine, The Norwegian University of Science and Technology (NTNU), Trondheim, Norway; 17 Helse Fonna, Haugesund, Norway; 18 Stavanger University Hospital, Stavanger, Norway; 19 Patient Representative, Oslo University Hospital, Oslo, Norway; 20 Akershus University Hospital, Lørenskog, Norway; 21 Innlandet Hospital Trust, Lillehammer, Norway; 22 Centre of Bioinformatics, Faculty of Mathematics and Natural Sciences, University of Oslo, Oslo, Norway; 23 Head of Section for Cancergenomics Section for Cancer Genomics, Haukeland University Hospital, Bergen, Norway; 24 Section for Cancergenomics, Department of Pathology, Stavanger University Hospital, Stavanger, Norway; 25 Dept of Oncology, Helse Møre and Romsdal Health Trust, Ålesund, Norway; 26 Department of Pediatric Medicine, Oslo University Hospital, Oslo, Norway; 27 Northern Trøndelag Trust, Levanger, Norway; 28 Department of Oncology, Haematology and Palliative Care, Lovisenberg Diaconal Hospital, Oslo, Norway; 29 Dept of Pathology, Førde Hospital Trust, Førde, Norway; 30 Department of Oncology, Vestfold Hospital Trust, Tønsberg, Norway; 31 Department of Oncology, Kalnes Hospital, Grålum, Norway; 32 Department of Pathology, Vestfold Hospital Trust, Tønsberg, Norway; 33 Department of Haematology, Oslo University Hospital, Tønsberg, Norway; 34 Dept of Oncology, Førde Hospital Trust, Førde, Norway; 35 Institute of Health and Society, Department of Health Management and Health Economics, University of Oslo, Oslo, Norway; 36 Division for Health Services, Norwegian Institute of Public Health, Oslo, Norway; 37 Department of Chemistry, Bioscience and Environmental Engineering, University of Stavanger, Stavanger, Norway; 38 Dept of Health Sciences, NTNU, Ålesund, Norway

**Keywords:** Precision medicine, Pan-cancer, Diagnostics, Mutations, Drugs

## Abstract

**Background:**

Matching treatment based on tumour molecular characteristics has revolutionized the treatment of some cancers and has given hope to many patients. Although personalized cancer care is an old concept, renewed attention has arisen due to recent advancements in cancer diagnostics including access to high-throughput sequencing of tumour tissue. Targeted therapies interfering with cancer specific pathways have been developed and approved for subgroups of patients. These drugs might just as well be efficient in other diagnostic subgroups, not investigated in pharma-led clinical studies, but their potential use on new indications is never explored due to limited number of patients.

**Methods:**

In this national, investigator-initiated, prospective, open-label, non-randomized combined basket- and umbrella-trial, patients are enrolled in multiple parallel cohorts. Each cohort is defined by the patient’s tumour type, molecular profile of the tumour, and study drug. Treatment outcome in each cohort is monitored by using a Simon two-stage-like ‘admissible’ monitoring plan to identify evidence of clinical activity.

All drugs available in IMPRESS-Norway have regulatory approval and are funded by pharmaceutical companies. Molecular diagnostics are funded by the public health care system.

**Discussion:**

Precision oncology means to stratify treatment based on specific patient characteristics and the molecular profile of the tumor. Use of targeted drugs is currently restricted to specific biomarker-defined subgroups of patients according to their market authorization. However, other cancer patients might also benefit of treatment with these drugs if the same biomarker is present. The emerging technologies in molecular diagnostics are now being implemented in Norway and it is publicly reimbursed, thus more cancer patients will have a more comprehensive genomic profiling of their tumour. Patients with actionable genomic alterations in their tumour may have the possibility to try precision cancer drugs through IMPRESS-Norway, if standard treatment is no longer an option, and the drugs are available in the study. This might benefit some patients. In addition, it is a good example of a public–private collaboration to establish a national infrastructure for precision oncology.

*Trial registrations* EudraCT: 2020-004414-35, registered 02/19/2021; ClinicalTrial.gov: NCT04817956, registered 03/26/2021.

## Background

Curative treatment options for patients with metastatic solid tumors are still rare. Most diagnostic groups have standardized algorithms for evidence-based treatment, and when the patients progress on standard-of-care, inclusion in clinical studies is an option.

Although personalized patient care is not a new concept, precision cancer medicine, based on use of molecular testing to identify targetable alterations, represents a major development in the field. Findings based on these technologies have led to new paradigms of cancer treatment [1–5].

A high number of advanced solid tumours (30–80%) display potentially “actionable” genomic variants [6–8]. However, the clinical benefit of targeting these remains largely anecdotal. Less than 7% of cancer patients were estimated to benefit from genome-guided anti-cancer therapies in the US in 2018 [9]. Other studies using genomic profiling approaches with individualized matching of molecular variant and drug, report low inclusion rate and modest overall rate of clinical benefit [10], although much higher than the first generation precision medicine trials in oncology [11–13]. This is probably due to increasing knowledge about actionable genes and driver mutations in tumour development, resistance mechanisms, a more refined diagnostic work-up and availability of new targeted therapeutics [14].

There is an increasing demand for more clinical studies exploring precision cancer treatment. Methods for more extended molecular profiling are available, and a considerable number of drugs are already approved on specific indications. However, these drugs are restricted to be used within the approved indication. Some drugs targeting a specific pathway or gene aberration, might be efficient in other subgroups of patients, not yet fully investigated in clinical trials.

IMPRESS-Norway (NCT04817956) is a national investigator-initiated clinical study. The aim is to enhance knowledge about molecular variant-drug matches and harmonize access to genomic testing and off-label use of cancer drugs in Norway. Thereby, patients with advanced cancer will have access to extended molecular diagnostics and putatively also treatment based on the tumour characteristics. The study will use a combined umbrella and basket design and a Simon two-stage model of expanding cohorts to establish potentially effective combinations of biomarker and drug [15, 16].

The IMPRESS-Norway study design is based on the DRUP trial (Drug Rediscovery Protocol) which has been ongoing in the Netherlands for five years [17]. They treat patients with targeted drugs based on a molecular profile, and outside of their current market authorisation. The initial results from the DRUP-trial reveal a clinical benefit of 34% in the first 215 patients beyond 16 weeks [17, 18]. According to the study report, 46% of the patients that were referred after local genomic testing were included in the trial. Both the reported inclusion rate and overall rate of clinical benefit were higher than other studies of tumour molecular profiling with matched targeted treatment [8, 19]. Based on the success of the DRUP-trial, similar studies have been initiated in other European countries including Denmark, Norway, Sweden, and Finland [20].

There is a lack of data on drug safety and efficacy for rare indications outside of the approved label for many cancer drugs. Through this clinical study, where key clinical outcomes are systematically collected, missing data will be provided, and also benefit some patients by offering a new treatment line based on molecular profiling.

## Methods/design

Oslo University Hospital is the sponsor of the study. All hospitals in Norway with an oncology or haematology care unit participate; recruiting patients for molecular profiling and providing study-specific treatment to patients included in a study cohort. Patient recruitment started in April 2021, and by March 7, 2022, 298 patients have been enrolled.

### Study objectives

There are two primary objectives of the study. The first is to describe the anti-tumour activity and toxicity of commercially available, targeted anti-cancer drugs used for treatment of patients with advanced malignancy that harbours a genomic or expression variant known to be a drug target or to predict sensitivity to a drug. This will be measured by percentage of patients included/treated in a cohort defined by molecular profile, drug and cancer subtype with disease control at 16 weeks of treatment (stable disease or better). Treatment-related grade ≥ 3 side effects and serious adverse events will be monitored. The second primary objective is to facilitate patient access to commercially available targeted anti-cancer drugs of potential efficacy for treatment of an advanced malignancy that harbours a genomic or protein expression variant known to be a drug target or to predict sensitivity to a drug.

The secondary study objectives are as follows: (1) To further describe tumour response to treatment; (2) To perform extensive and longitudinal biomarker analyses, including (but not limited to) next generation sequencing [including whole genome and transcriptome sequencing (WGS and WTS respectively)], on a fresh tumour biopsy specimen and liquid biopsies (blood samples, effusions); (3) To map the patient journey through the Precision Cancer Medicine pipeline; and (4) To assess the availability of tumour tissue biopsy across and within tumour types.

The exploratory objectives are to study mechanisms of resistance by the use of serial fresh tumour biopsies for WGS/WTS and liquid biopsies; to evaluate clinical utility of circulating tumour DNA (ctDNA) in treatment decision procedures and in monitoring treatment response; to investigate response evaluation in cohorts with immunotherapy; to provide long term follow-up data on the patients; to investigate patient reported outcome measurements (including health-related quality of life); to investigate cost-effectiveness; to determine clinical course in patients not included in treatment cohorts; to explore other methods for response evaluation (Artificial Intelligence (AI) in radiology for instance); to investigate need for medical genetics expertise for follow-up of patients; and to investigate the ESMO Scale for Clinical Actionability of molecular Targets (ESCAT) guidelines for actionable targets.

### Study design

This is a prospective, non-randomized, open-label combined basket- and umbrella clinical study based on a Simon two stage model of expanding cohorts. This model, tested in the Targeted Agent and Profiling Utilization Registry (TAPUR) and DRUP studies, has been designed to effectively test a set of drugs using a precision medicine algorithm while minimizing the number of patients required [21].

Each tumour type/molecular variant/drug will define a specific cohort, and each cohort constitutes a Simon two stage trial model to identify cohorts with evidence of clinical activity. Stage 1 cohorts will enrol eight participants and will be considered positive if ≥ 1 show objective response or stable disease at 16 weeks of treatment. In case of a positive stage 1, a stage 2 will be initiated enrolling 16 additional participants into the cohort. If no patients experience disease control at week 16 (in stage 1), the cohort will be closed. Four or fewer responses out of 24 in the stage 2 cohort, will suggest a lack of activity, while five or more responses will suggest that further investigation of the drug in the tumour/variant cohort is warranted. For positive stage 2 cohorts, a stage 3 expansion cohort of up to 130 additional patients may be opened following agreements between the trial and the company providing the drug. Such expansion cohorts are organised like a phase 2 trial and as of February 2022, the four Regional Health Authorities in Norway have jointly decided that reimbursement of drug may be granted at the request of the trial for specific cohorts and for responding patients following evaluation at 16 weeks according to a pay for performance model. This in line with what has been set out in The Netherlands for the DRUP trial (PMID: 31038154).

For purposes of cohort definition, the ‘variant’ category can be defined at the level of the gene that harbours the mutation or overexpression (for instance HER2) or be determined by specific mutations (like BRAF V600E) or represent a profile (such as tumour mutational burden; TMB). Pan-cancer cohorts will be defined when the molecular subtype is very rare [15, 16].

### Available drugs in IMPRESS-Norway

Patients included for treatment in IMPRESS-Norway are treated with commercially available licenced drugs provided by participating pharmaceutical companies. This is a dynamic study design and will include more drugs as more pharmaceutical companies are participating. For every available drug though, additional drug-specific study information will be provided in separate amendments (drug-specific study manuals). These include the Summary of Product Characteristics (SmPC) and/or Investigator's Brochure (IB), drug-specific patient information in Norwegian, and a drug-specific study manual including drug-specific inclusion and exclusion criteria, risk benefit assessment and treatment schedules. Currently we have available a PDL1 inhibitor and several targeted therapies. Eight drugs were available (from Roche) when initiating the trial and new agreements have since been made with Novartis, Eli-Lilly and Incyte, providing additional drugs. We have also defined cohorts repurposing “old” generic drugs, which comes with a low cost, and covered by research funding (4 cohorts with 8 patients each).

### Combination

Drugs used in the study can either be used as monotherapy or as combinations approved by US Food and Drug Administration (FDA) or the European Medicine Agency (EMA). Each cohort will include one specific drug or one drug combination (approved) in one diagnostic subgroup. In selected cohorts, a study drug can be added to a standard backbone of systemic treatment after approvals from the involved pharmaceutical company and the Norwegian Medicines Agency.

### Justification of dose

Initial drug dosing, dose modifications and management of drug-related toxicities will be according to the U.S. Food and Drug Administration (FDA) and/or European Medicines Agency (EMA) approved label (or under revision for approval) and/or manufacturer’s recommendations. If several doses are described, considerations will be made by the study team to select dosing based on current knowledge. Details on mechanism of action, potential risks and benefits, and considerations on concomitant medication are included in the protocol or the drug specific manuals with reference to the specific SmPC/IB.

### Patient selection and procedures

Patients with advanced malignancies are eligible after disease progression on standard treatment. Two informed consents are obtained, first for the molecular screening and, if the patient is eligible for treatment phase, the second drug specific consent for the specific drug available in the study for that molecular subgroup. Children are currently only eligible for the molecular screening.

**Fig. 1:**
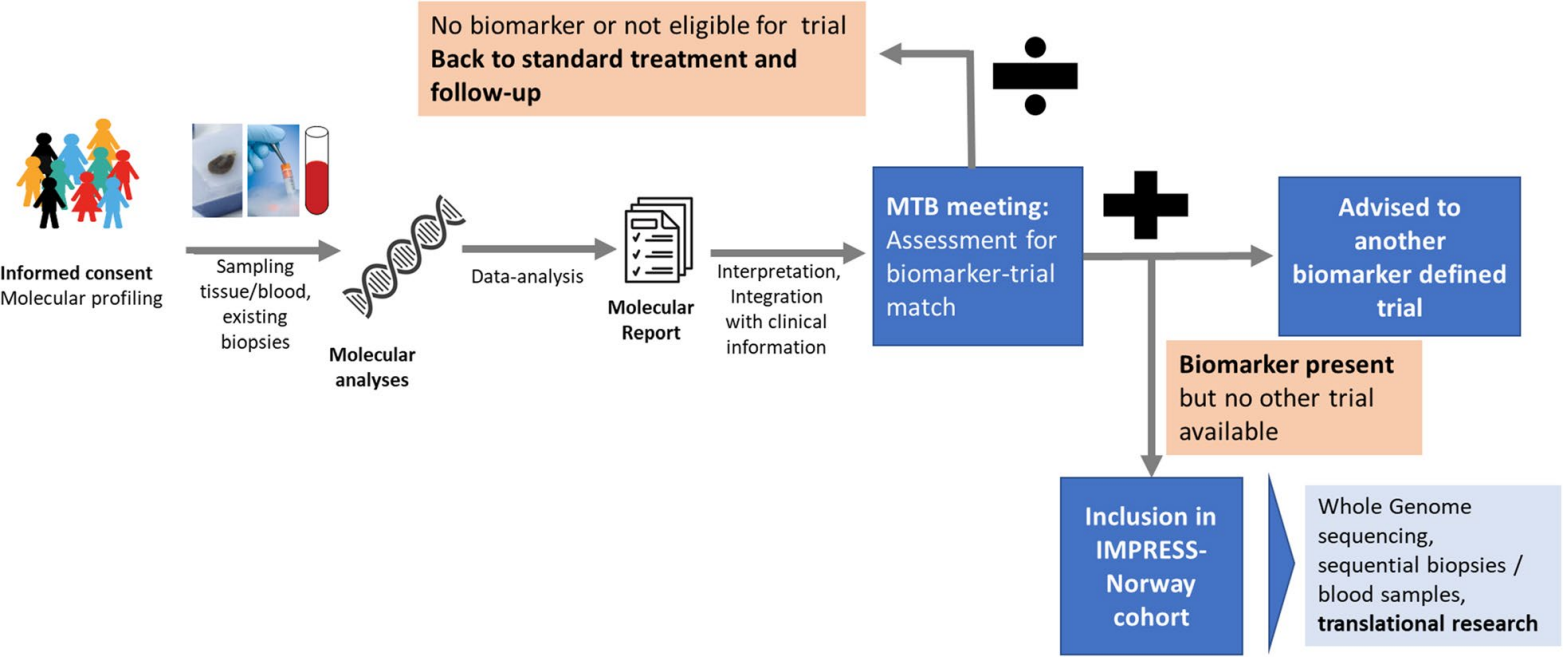
Flow chart of patient inclusion. Patients sign informed consent prior to the molecular screening. If offered treatment in a cohort, the patients sign a drug specific informed consent before initiating treatment

The treating physician will determine whether a patient eligible for comprehensive molecular profiling meets all the general inclusion criteria and none of the exclusion criteria for participation in the molecular profiling phase and collect the first informed consent form (molecular profiling ICF). Two Norwegian university hospitals have established comprehensive molecular profiling—and the remaining four university hospitals are underway with establishing such diagnostics. This as part of the publicly funded Infrastructure for Precision Diagnostics—cancer (InPreD Norway). The comprehensive molecular profiling includes genomic analyses using the TruSight Oncology 500 panel (TSO500) by Illumina as the initial standard (now reimbursed as part of the health care in Norway) which can be supplemented with immunohistochemistry (IHC), fluorescence in situ hybridisation (FISH) or necessary molecular/diagnostic tests. The treating physician will submit a biopsy specimen for molecular profiling through their local pathology department according to guidelines. In addition, a plasma sample for trial-specific molecular testing (i.e. liquid biopsy) will be submitted to an approved trial laboratory. In January 2022, two of the university hospitals are approved trial laboratories, and the remaining university hospitals are planned ready within the next six months. If no tumour material is available, a patient can be included based on the liquid biopsy analysis alone. All study specific procedures must take place after signing the ICF. Plasma samples will also be collected and analysed for molecular alterations in ctDNA. The test results will be submitted to the national molecular tumour board and discussed together with the clinical data provided by the treating physician. The national molecular tumour board can advise on experimental treatment opportunities (i.e. available trials and compassionate use programs) including IMPRESS-Norway cohort inclusion. If one or more molecular variant fulfils the criteria defined for an available drug, the national molecular tumour board will advise a possible treatment in an IMPRESS-Norway cohort, as described below. If all drug specific eligibility criteria are confirmed, the patient can consent to treatment with the proposed drug and sign the drug-specific informed consent (see also Fig. 1 ‘Schematic overview of patient submission’). If an ongoing clinical study other than IMPRESS-Norway is available for the patient, recommendations for the patient will be made based on what may benefit the patient most.

### Selected inclusion and exclusion criteria—molecular diagnostics screening phase

Selected inclusion criteria

1. ECOG performance status 0–2.
2. Patients must have measurable or evaluable disease. RECIST v1.1 [22, 23] will be used for patients with solid tumours. For patients with multiple myeloma or non-Hodgkin lymphoma, International Myeloma Working Group (IMWG) response criteria [24] and CHESON/Lugano guidelines [25] will be used, respectively. For glioblastoma patients, Response Assessment in Neuro-Oncology (RANO) criteria will be used [26]. iRECIST will be used for immunotherapy-cohorts. European LeukemiaNet recommendations for diagnosis and management of acute myeloid leukaemia (ELN-AML) response criteria will be used accordingly.
  - Patients whose disease cannot be objectively measured by physical or radiographic examination (e.g., elevated serum tumour marker only) are NOT eligible, except for cancer antigen-125 (CA-125) for ovarian cancer and prostate-specific antigen (PSA) for prostate cancer [27].
3. The patient is, in the opinion of the investigator, a candidate for a treatment cohort in IMPRESS Norway or another clinical study in Norway
4. Ability to understand and the willingness to sign a written informed consent

### National molecular tumour board and treatment plan

A national molecular tumour board is established as part of the public health care system and evaluate patients where comprehensive genomic profiling has been performed. They consider each molecular profile and the potential benefit of treatment with a matched drug. The national molecular tumour board is organized as a virtual meeting using a secure platform and is staffed by regular and ad hoc members nationwide, including experts in several fields, such as clinical oncology, haematology, gynaecological oncology, pathology, medical genetics, cancer biology, bioinformatics, and pharmacology. The treating physician will participate, in addition to health personnel at the relevant sites. Clinical patient characteristics presented by the treating physician include age, sex, performance status and relevant medical history. As beforementioned, possible outcomes of the review may be a proposal of treatment in a cohort in the IMPRESS-Norway study, recommendation for a different clinical study, or no available clinical studies and referral to standard treatment and follow-up. New study cohorts in IMPRESS-Norway depend on approval by the National Principal Investigator. The treatment recommendation is entered in the study database.

### Recruitment and consent

Patients will be recruited by the participating 17 hospital trusts. Prior to obtaining informed consent for molecular screening, the patient will be provided with information about molecular testing and evaluation by the national molecular tumour board (Patient Information Sheet for pre-inclusion testing—Molecular Profiling ICF). Patients found eligible for the treatment will be provided with written information on specific study drug and cohort (Patient Information Sheet for a specific clinical cohort in the trial—Drug ICF) before obtaining the second consent for entering treatment phase of the trial. The written informed consent (voluntarily) is obtained prior to any study specific procedures, including specific screening procedures. Patients have the right to withdraw from the study at any time, without giving an explanation and without consequences for subsequent care.

All patients also consent to collection of patient-reported outcome measurements (PROMS) and to coupling with data from Norwegian health registers (Cancer Registry of Norway, Norwegian Patient Registry, Norwegian Prescription Database, Primary care patient- and user Register (KPR) and Statistics Norway for socioeconomic characteristics).

### Suspected germline alteration

If the molecular analysis indicates a germ line alteration as defined by ESMO guidelines and the patient might benefit from genetic counselling, the patient can choose to be referred to a medical geneticist for such counselling. This is outlined in the patient informed consent form [28].

### Treatment at local hospitals

Patients will be treated at their local hospital, with 17 hospitals participating. The schedule of study activities is shown in Table 1 (molecular profiling) and Table 2 (treatment phase). Only HTA-approved drugs are provided, and typical toxicities will be recognised and manageable at hospitals that usually treat cancer patients. However, as many of these hospitals lack expertise in running clinical studies, and the university hospitals will provide guidance and assistance for study personnel, if needed. Children are currently not included in treatment cohorts.

**Table 1:** Schedule of Activities (SoA) for molecular profiling

Study procedures	Molecular profiling D1	W16^a^	Survival FU
Informed consent for molecular profiling	X		
Check inclusion criteria and exclusion criteria for molecular profiling	X		
ECOG PS	X		
Survival^a^	X	X	Through cancer registry
QoL^a^	X	X	
Central lab sampling	X		
Tumor biopsy	X		
Plasma /serum samples	X		

^a^Patients not enrolled in the treatment phase are followed-up W16 for survival and QoL

**Table 2:** Schedule of Activities (SoA) for patients enrolled in treatment-cohort

Study procedures	Screening Treatment cohort	Treatment phase						EOT	Survival FU
	(D 1–21)	D1^g^	W8^g^	W16^g^	W26^g^	W39^h^	QW13^h^		Q26W for 2 years after end of treatment
Check inclusion criteria and exclusion criteria for treatment phase + Drug specific selection criteria	X								
Informed consent^a^	X								
Medical history	X								
Drug dispensing^b^		X	X	X	X	X	X		
Physical examination	X		X	X	X	X	X	X	
Vital signs and ECOG PS	X	X	X	X	X	X	X	X	
ECG	X								
AE/SAE assessment	X		X	X	X	X	X	X	
Concomitant medication	X		X	X	X	X	X	X	
QoL (V)	X			X	X	X	X	X	
Laboratory Assessments^c^	X		X	X	X	X	X	X	
Pregnancy test^d, e^	X	Monthly as long as use of contraception is required, see drug specific amendment							
Central lab sampling	X								
Plasma/serum samples	X		X	X	X	X	X	X	
Urine^i^	X		X					X	
Feces^i^	X		X						
Tumor biopsy	X			X				X	
Other material^f^	X		X	X				X	
Tumor assessment	X		X	X	X	X	X	X	
Survival follow-up									X

^a^Drug specific informed consent

^b^Treatment according to drug specific manuals

^c^Patients should be routinely monitored for serum creatinine and electrolytes (including magnesium) while on therapy. Liver function (including AST and ALT) should be monitored monthly during the first 6 months of treatment, and as clinically indicated thereafter.

^d^Less than 72 h before treatment

^e^If patient is sexual abstinent, there is no need for pregnancy testing, but the patient must confirm abstinence monthly

^f^Pleural, effusion/ascites collected when possible/available

^g^Visit window +/− 7 days, year 1.

^h^Visit window +/− 14 days after year 1.

^i^Selected cohorts only

### Selected inclusion criteria—treatment phase

To enter the treatment phase of the trial, both the above and the following criteria must apply; other drug specific criteria may be added in the Drug Specific Amendment.

1. Patient with a pathology-proven non-curable malignant disease who is no longer benefitting from standard treatment or for whom, in the opinion of the investigator, no such treatment is available or indicated.
2. Patients must have acceptable organ function as defined below (some specifics for hematologic diagnoses):
  - Absolute neutrophil count ≥ 1.5 × 10^9^ /L
  - Hemoglobin ≥ 9 g/dl
  - Platelets ≥ 75,000/µl
  - Total bilirubin < 1.5 × institutional upper limit of normal (ULN)
  - Aspartate aminotransferase (AST) (serum glutamic-oxaloacetic transaminase [SGOT]) and alanine aminotransferase (ALT) (serum glutamic-pyruvic transaminase < 2.5 × institutional upper limit of normal (ULN) (or < 5 × ULN in patients with known hepatic metastases)
  - Calculated or measured creatinine clearance ≥ 40 mL/min/1.73 m^2^
3. For orally administered drugs, the patient must be able to swallow and tolerate oral medication and must have no known malabsorption syndrome.
4. Results must be available from a genomic / molecular test performed in a preapproved laboratory. The test used to qualify a patient for participation in IMPRESS-Norway may have been performed on any specimen of the patient’s tumour obtained at any point during the patient’s care at the discretion of the patient’s treating physician. Genomic assays performed on cell-free DNA in plasma (“liquid biopsies”) will also be acceptable. Information from these analyses might be used upon progression, for evaluation of possible new cohort-inclusion.
5. Have a genomic profile indicating that treatment with one of the anti-cancer therapies included in this study may have potential clinical benefit
6. Women of child-bearing potential and men must agree to use adequate highly effective methods of contraception for the duration of study participation
7. Female participants must have a negative highly sensitive pregnancy test < 1 month prior to inclusion.
8. Male patients should avoid impregnating a female partner.
9. Ability to understand and the willingness to sign a written informed consent

### Selected exclusion criteria—treatment phase

A potential participant who meets any of the following criteria for medical conditions will be excluded from inclusion in the molecular profiling.

1. Patients with the following pre-existing cardiac diagnosis, uncontrolled angina, uncontrolled atrial or ventricular arrhythmias, or symptomatic congestive heart failure.
2. Patients with left ventricular ejection fraction (LVEF) known to be < 40%.
3. Patients with any other clinically significant medical condition which, contradicts participation in the study.
4. Patients with known progressive brain metastases determined by serial imaging or declining neurologic function in the opinion of the treating physician. Patients with previously treated/stable brain metastases are eligible.
5. Patients eligible to enter other ongoing trials which have the potential to benefit the patients equally or more than an IMPRESS-Norway cohort, and for whom access to the ongoing trials is manageable.
6. Ongoing toxicity > Common Terminology Criteria for Adverse Events (CTCAE) grade 2, other than peripheral neuropathy, related to anti-tumour treatment that was completed within 4 weeks prior to treatment initiation. Patients with ongoing peripheral neuropathy of ≥ CTCAE grade 3.
7. Patients with stroke (including transient ischemic attack (TIA)) or acute myocardial infarction within 4 months before the first dose of study treatment
8. If the patient’s tumour has a genomic variant known to confer resistance to an anti-cancer agent available in this study, the patient will not be eligible to receive that agent but will be eligible to receive other drugs available in this study if all inclusion and exclusion criteria are met for that drug
9. Patient is receiving any other anti-cancer therapy (cytotoxic, biologic, radiation, or hormonal other than for replacement) except for medications that are prescribed for supportive care but may potentially have an anti-cancer effect (e.g., megestrol acetate, bisphosphonates) or ongoing castration-intent therapy for prostate cancer. These medications must have been started ≥ 1 month prior to enrolment on this study. Patients may be on warfarin, low molecular weight heparin or direct factor Xa inhibitors.

Note: For each drug included in this protocol, specific inclusion and exclusion criteria (based on the Summary of Product Characteristics (SPC) or manufacturer’s recommendations) may also apply.

## Collateral research

### Sample collection/biobanking

The collection of biological samples for translational and biomarker research is an important part of this study. Tumour samples will be collected up to at three different time points (prior to treatment, during treatment (at 16 weeks) and upon progression if not progression at or before 16 weeks). Plasma samples will be collected at evaluation timepoints, and pleura effusion/ascites will be collected if available.

Whole genome sequencing and RNA-sequencing will be performed on tumour material from before treatment, after 16 weeks (if on treatment) of treatment and upon progression. This might inform on mechanisms of response or resistance. As a part of the molecular profiling, ctDNA analyses will be performed for the first 500 patients by FoundationOne® Liquid CDx provided by Foundation Medicine Inc. This will allow for comparison between liquid biopsy and tumour biopsy. In addition, Foundation Medicine provides tests for 150 patients lacking available tumour material. For the subsequent 500 patients included in IMPRESS-Norway, ctDNA analyses will be performed inhouse and provided by Illumina (TSO500 ctDNA test). The biologic material will be stored in the IMPRESS-Norway-biobank and the data will be stored in TSD (Services for Sensitive Data). In addition, several analyses will be done on material from the different cohorts.

### International collaboration

It is anticipated that many cohorts will represent small subgroups. Patients from the Netherlands, Denmark, Sweden, Finland and most likely several other European countries will be included in similar but independent protocols. We plan to merge data from these protocols to ensure sufficient patient numbers for analyses of efficacy in every cohort. A large network of these studies is being established in Europe, and funding is in place from the Nordic Trial Alliance for coordination and aggregation on clinical outcome data [20].

For drugs expected to be effective in a pan-tumour-type manner, cohorts of the same drug and tumour profile may be analysed jointly.

### Statistical analyses

Admissible designs lie between MiniMax and Optimal designs and fits well for both (i.e. small maximum sample size, and low expected sample size under the null hypothesis of low activity). A true response rate (complete response [CR], partial response [PR], stable disease [SD]) of less than 10% will be considered of no clinical interest. A response rate of 30% or more will be considered of sufficient interest to warrant further study in a confirmatory trial.

Negative cohorts (no patients benefitting) will be closed. If one or more of the first eight patients benefit from treatment, an additional 16 participants who fulfil the inclusion criteria will be included in the cohort. Four or fewer responses out of 24 included in total, will suggest a lack of activity, while five or more responses will suggest that further investigation of the drug in the tumour/variant cohort is warranted. This monitoring rule has 85% power and an alpha error rate of 7.8%. These operating characteristics were selected to represent a reasonable compromise between high power, low false positive rates, and desire for small sample sizes.

When an individual cohort terminates accrual early or completes accrual, efficacy, and toxicity data in addition to patient characteristics will be summarised descriptively with tabulations, rates and confidence intervals.

### Health economics, cost-effectiveness analyses (CEA) and national registries

Health Economics data, associated with medical encounters, will be collected by the investigator and study-site personnel for all participants (both participants in a cohort and those not included in a treatment cohort) throughout the study. In addition, clinical data will be supplemented by information from several registers: Cancer Registry of Norway, Norwegian Patient Registry, Norwegian Prescription Database, Primary care patient- and user Register (KPR) and Statistics Norway for socioeconomic characteristics. Protocol-mandated procedures, tests, and encounters are excluded. Health economics research will go on throughout the duration of the IMPRESS trial and will be coordinated with the more general discussion on novel implementation methods for Precision Cancer Medicine (PCM), health technology assessments (HTA) of PCMs and data structure.

From the perspective of the Norwegian health care system, several analyses will be performed. To conduct cost-effectiveness analyses, costs and health outcomes of target drug treatment will be compared to the patient group eligible for genetic testing, but where no relevant target agents are identified. During the first years of IMPRESS-Norway, the comparator will contain an aggregated group of patients with several indications, but this group will gradually be refined to match the treated patients (for instance by indication, other condition specific characteristics, sex and age).

Costs will include costs related to genetic testing (testing, device, analysis, tumour board etc.), treatment, adverse events, follow-up and additional treatment (specialist and primary care), primary care (practical assistance and institutions), and best supportive care.

### Medical genetics

For patients receiving treatment in the study, patient tumour material will be analysed by WGS, and germ line will be analysed for comparison. We might detect germline alterations indicating increased risk for developing disease, but the frequencies of these are unknown. In the era of personalized cancer care (or precision oncology), this might be an issue that needs focus in the coming years. Inheritable disease risk will be explored in our study, and the departments of medical genetics in Norway will be consulted through a defined pipeline.

## Discussion

In this study, we will evaluate clinical benefit of approved drugs outside current indication based on extended molecular testing of cancers available through the public health care system in Norway. Patients with a specific cancer subtype and molecular alterations in their tumour cells matching the drugs in the study, will define a cohort and receive a potentially effective treatment. The study is open to patients with advanced malignant disease after progression on standard treatment. The patients will be treated at their local hospital to ensure knowledge transfer throughout the health care system.

Extensive collection of biological material (including biopsy preferably at three time points, before treatment, during treatment and upon disease progression) and in-depth molecular characterisation including WGS, WTS and ctDNA-analyses, will provide new knowledge regarding response and resistance to treatment. The biobank and data will be available for research groups in Norway pending permission for specific research projects- and after evaluation in the data/material committee. This will facilitate state-of-the-art translational research e.g. immune microenvironment studies, drug sensitivity analyses (selected cohorts), immune cell analyses.

The rationale and design of the IMPRESS-Norway trial is similar to other precision cancer medicine- trials (DRUP-related trials) recently launched in Europe and North America. Data sharing within this network in Europe is planned in order to aggregate data on small patient cohorts. This is important because of the expected low number of eligible patients for some cohorts [17]. In addition, long-term follow up data using the Cancer Registry of Norway as well as additional national relevant registers, will be collected on all patients screened in IMPRESS-Norway. A unique advantage for the Nordic countries is the access to long-term follow up data on both treated and non-treated patients through the cancer registries. These data allow for a richer variety in model development compared to other countries.

IMPRESS-Norway (https://impressnorway.com) is an academic clinical trial with public support and industry funding through sourcing of free drugs and support per included patient with its own budget and agreements. The trial applies a new public infrastructure for molecular cancer diagnostics (InPreD) which includes a national molecular tumour board that serve IMPRESS-Norway as well as other molecular based clinical trials in oncology. The IMPRESS-Norway was formally opened April 1, 2021, and has screened 298 cancer patients per March 7, 2022. 59 patients have been allocated treatment.

## Data Availability

Not applicable—no data presented.

## Abbreviations

AI: Artificial intelligence
ALT: Alanine aminotransferase
AST: Aspartate aminotransferase
CA-125: Cancer antigen-125
CEA: Cost-effectiveness analyses
CR: Complete response
CSF: Cerebrospinal fluid
CTCAE: Common Terminology Criteria for Adverse Events
ctDNA: Circulating tumour DNA
DRUP: Drug Rediscovery Protocol
ECOG: Eastern Cooperative Oncology Group
ELN-AML: European LeukemiaNet recommendations for diagnosis and management of acute myeloid leukemia
EMA: European Medicines Agency
ESCAT: ESMO Scale for Clinical Actionability of molecular Targets
FISH: Fluorescence in situ hybridisation
HTA: Health technology assessment
HTS: High-throughput sequencing
IB: Investigator's brochure
ICF: Informed consent form
IHC: Immunohistochemistry
IMWG: International Myeloma Working Group
IMPRESS-Norway: Improving public cancer care by implementing precision medicine in Norway
InPreD: Infrastructure for Precision Diagnostics
LVEF: Left ventricular ejection fraction
NGS: Next generation sequencing
PCM: Patient care management
PR: Partial response
PSA: Prostate-specific antigen
QoL: Quality of life
RANO: Response Assessment in Neuro-Oncology
SD: Stable disease
SGOT: Serum glutamic-oxaloacetic transaminase
SGPT: Serum glutamic-pyruvic transaminase
SoA: Schedule of Activities
SPC: Summary of product characteristics
TAPUR: Targeted Agent and Profiling Utilization Registry
TIA: Transient ischemic attack
ULN: Upper limit of normal
U.S. FDA: U.S. Food and Drug Administration
WGS: Whole genome sequencing
WTS: Whole transcriptome sequencing

## References

[1] Garraway LA (2013). Genomics-driven oncology: framework for an emerging paradigm. J Clin Oncol.

[2] Lawrence MS (2014). Discovery and saturation analysis of cancer genes across 21 tumour types. Nature.

[3] Macconaill LE, Garraway LA (2010). Clinical implications of the cancer genome. J Clin Oncol.

[4] Rafii A (2014). Where cancer genomics should go next: a clinician's perspective. Hum Mol Genet.

[5] Andre F (2014). Comparative genomic hybridisation array and DNA sequencing to direct treatment of metastatic breast cancer: a multicentre, prospective trial (SAFIR01/UNICANCER). Lancet Oncol.

[6] Von Hoff DD (2010). Pilot study using molecular profiling of patients' tumors to find potential targets and select treatments for their refractory cancers. J Clin Oncol.

[7] Jardim DL (2015). Impact of a biomarker-based strategy on oncology drug development: a meta-analysis of clinical trials leading to FDA approval. J Natl Cancer Inst.

[8] Le Tourneau C (2015). Molecularly targeted therapy based on tumour molecular profiling versus conventional therapy for advanced cancer (SHIVA): a multicentre, open-label, proof-of-concept, randomised, controlled phase 2 trial. Lancet Oncol.

[9] Marquart J, Chen EY, Prasad V (2018). Estimation of the percentage of US patients with cancer who benefit from genome-driven oncology. JAMA Oncol.

[10] Rodon J (2019). Genomic and transcriptomic profiling expands precision cancer medicine: the WINTHER trial. Nat Med.

[11] Ree AH (2020). Molecularly matched therapy in the context of sensitivity, resistance, and safety; patient outcomes in end-stage cancer—the MetAction study. Acta Oncol.

[12] Flaherty KT (2020). Molecular landscape and actionable alterations in a genomically guided cancer clinical trial: National Cancer Institute Molecular Analysis for Therapy Choice (NCI-MATCH). J Clin Oncol.

[13] Johnson A (2017). Clinical use of precision oncology decision support. JCO Precis Oncol.

[14] Li Y (2020). Patterns of somatic structural variation in human cancer genomes. Nature.

[15] Simon R (1989). Optimal two-stage designs for phase II clinical trials. Control Clin Trials.

[16] Jung SH (2004). Admissible two-stage designs for phase II cancer clinical trials. Stat Med.

[17] van der Velden DL (2019). The Drug Rediscovery protocol facilitates the expanded use of existing anticancer drugs. Nature.

[18] van de Haar J, Hoes L, Voest E (2019). Advancing molecular tumour boards: highly needed to maximise the impact of precision medicine. ESMO Open.

[19] Tsimberidou AM (2017). Initiative for molecular profiling and advanced cancer therapy (IMPACT): an MD Anderson precision medicine study. JCO Precis Oncol.

[20] NKI. European collaboration. The Netherlands Cancer Institute, Press Release, Nov 26, 2021: “Dutch-Nordic Alliance for Precision Cancer Medicine launched”. 2021. https://www.nki.nl/news-events/news/dutch-nordic-alliance-for-precision-cancer-medicine-launched/.

[21] Mangat PK (2018). Rationale and Design of the Targeted Agent and Profiling Utilization Registry (TAPUR) Study. JCO Precis Oncol..

[22] Eisenhauer EA (2009). New response evaluation criteria in solid tumours: revised RECIST guideline (version 1.1). Eur J Cancer.

[23] Seymour L (2017). iRECIST: guidelines for response criteria for use in trials testing immunotherapeutics. Lancet Oncol.

[24] Rajkumar SV (2014). International Myeloma Working Group updated criteria for the diagnosis of multiple myeloma. Lancet Oncol.

[25] Cheson BD (2014). Recommendations for initial evaluation, staging, and response assessment of Hodgkin and non-Hodgkin lymphoma: the Lugano classification. J Clin Oncol.

[26] Wen PY (2010). Updated response assessment criteria for high-grade gliomas: response assessment in neuro-oncology working group. J Clin Oncol.

[27] Rustin GJ (2011). Definitions for response and progression in ovarian cancer clinical trials incorporating RECIST 1.1 and CA 125 agreed by the Gynecological Cancer Intergroup (GCIG). Int J Gynecol Cancer.

[28] Mandelker D (2019). Germline-focussed analysis of tumour-only sequencing: recommendations from the ESMO Precision Medicine Working Group. Ann Oncol.

