# Correction to: Improving public cancer care by implementing precision medicine in Norway: IMPRESS-Norway

**DOI:** 10.1186/s12967-022-03518-0

**Published:** 2022-07-15

**Authors:** Åslaug Helland, Hege G. Russnes, Gro Live Fagereng, Khalid Al-Shibli, Yvonne Andersson, Thomas Berg, Line Bjørge, Egil Blix, Bodil Bjerkehagen, Sigmund Brabrand, Marte Grønlie Cameron, Astrid Dalhaug, Dalia Dietzel, Tom Dønnem, Espen Enerly, Åsmund Flobak, Sverre Fluge, Bjørnar Gilje, Bjørn Tore Gjertsen, Bjørn Henning Grønberg, Kari Grønås, Tormod Guren, Hanne Hamre, Åse Haug, Daniel Heinrich, Geir Olav Hjortland, Eivind Hovig, Randi Hovland, Ann-Charlotte Iversen, Emiel Janssen, Jon Amund Kyte, Hedda von der Lippe Gythfeldt, Ragnhild Lothe, Jo-Åsmund Lund, Leonardo Meza-Zepeda, Monica Cheng Munthe-Kaas, Olav Toai Duc Nguyen, Pitt Niehusmann, Hilde Nilsen, Katarina Puco, Anne Hansen Ree, Tonje Bøyum Riste, Karin Semb, Eli Sihn Samdal Steinskog, Andreas Stensvold, Pål Suhrke, Øyvind Tennøe, Geir E. Tjønnfjord, Liv Jorunn Vassbotn, Eline Aas, Kristine Aasebø, Kjetil Tasken, Sigbjørn Smeland

**Affiliations:** 1 Institute for Cancer Research/Department of Oncology/Division of Cancer Medicine, Oslo University Hospital, Oslo, Norway; 2 Institute of Clinical Medicine, University of Oslo, Oslo, Norway; 3 Department of Pathology, Oslo University Hospital, Oslo, Norway; 4 Dept. of Pathology, Nordland Hospital, Bodø, Norway; 5 Hospital Pharmacies Enterprise, Oslo, Norway; 6 Department of Pathology, University Hospital in North of Norway, Tromsø, Norway; 7 Department of Clinical Medicine, UiT The Arctic University of Norway, Tromsø, Norway; 8 Haukeland University Hospital, Bergen, Norway; 9 Centre for Cancer Biomarkers CCBIO, Department of Clinical Science, University of Bergen, Bergen, Norway; 10 Department of Oncology, University Hospital in North of Norway, Tromsø, Norway; 11 Hospital of Southern Norway, Kristiansand, Norway; 12 Department of Oncology and Palliative Medicine, Nordland Hospital Trust, Bodø, Norway; 13 Telemark Hospital Trust, Skien, Norway; 14 Department of Research, The Cancer Registry of Norway, Oslo, Norway; 15 Department of Oncology, The Cancer Clinic, St Olavs Hospital, Trondheim University Hospital, Trondheim, Norway; 16 Department of Clinical and Molecular Medicine, The Norwegian University of Science and Technology (NTNU), Trondheim, Norway; 17 Helse Fonna, Haugesund, Norway; 18 Stavanger University Hospital, Stavanger, Norway; 19 Patient Representative, Oslo University Hospital, Oslo, Norway; 20 Akershus University Hospital, Lørenskog, Norway; 21 Innlandet Hospital Trust, Lillehammer, Norway; 22 Centre of Bioinformatics, Faculty of Mathematics and Natural Sciences, University of Oslo, Oslo, Norway; 23 Head of Section for Cancergenomics Section for Cancer Genomics, Haukeland University Hospital, Bergen, Norway; 24 Section for Cancergenomics, Department of Pathology, Stavanger University Hospital, Stavanger, Norway; 25 Dept of Oncology, Helse Møre and Romsdal Health Trust, Ålesund, Norway; 26 Department of Pediatric Medicine, Oslo University Hospital, Oslo, Norway; 27 Northern Trøndelag Trust, Levanger, Norway; 28 Department of Oncology, Haematology and Palliative Care, Lovisenberg Diaconal Hospital, Oslo, Norway; 29 Dept of Pathology, Førde Hospital Trust, Førde, Norway; 30 Department of Oncology, Vestfold Hospital Trust, Tønsberg, Norway; 31 Department of Oncology, Kalnes Hospital, Grålum, Norway; 32 Department of Pathology, Vestfold Hospital Trust, Tønsberg, Norway; 33 Department of Haematology, Oslo University Hospital, Tønsberg, Norway; 34 Dept of Oncology, Førde Hospital Trust, Førde, Norway; 35 Institute of Health and Society, Department of Health Management and Health Economics, University of Oslo, Oslo, Norway; 36 Division for Health Services, Norwegian Institute of Public Health, Oslo, Norway; 37 Department of Chemistry, Bioscience and Environmental Engineering, University of Stavanger, Stavanger, Norway; 38 Dept of Health Sciences, NTNU, Ålesund, Norway

## Correction to: Journal of Translational Medicine (2022) 20:225 https://doi.org/10.1186/s12967-022-03432-5

Following publication of the original article [1], we have been notified that two authors’ names and affiliations were incorrectly mentioned. They should be as follows:

First name—Hilde, family name—Nilsen^2,3^

First name—Katarina, family name—Puco^1,28^

The original article has been corrected.
